# Prevalence of iron and folic acid supplements consumption and associated factors among pregnant women in Eswatini: a multicenter cross-sectional study

**DOI:** 10.1186/s12884-021-03881-8

**Published:** 2021-06-30

**Authors:** Gugulethu N. Mabuza, Alexander Waits, Owen Nkoka, Li-Yin Chien

**Affiliations:** 1 International Health Program, National Yang Ming Chiao Tung University, 155 Li-Nong Street, Section 2, Bei-Tou, 11221 Taipei, Taiwan; 2 Institute of Public Health, National Yang Ming Chiao Tung University, Taipei, Taiwan; 3 Taoyuan General Hospital, Ministry of Health and Welfare, Taoyuan, Taiwan; 4 Institute of Health and Wellbeing, University of Glasgow, Glasgow, UK; 5 Institute of Community Health Care, College of Nursing, National Yang Ming Chiao Tung University, 155 Li-Nong Street, Section 2, 11221 Taipei, Taiwan

**Keywords:** Iron and folic acid supplements, Associated factors, Pregnant women, Eswatini

## Abstract

**Background:**

During pregnancy, nutritional requirements increase and if not met, pregnancy-related complications may manifest. To prevent these undesirable outcomes, the World Health Organization recommends daily oral iron and folic acid (IFA) supplementation as part of antenatal care. Despite this recommendation, the use of IFA supplements is still very low in several developing countries. Additionally, no prior information exists regarding the level of consumption of IFA in Eswatini. Thus, this study aimed to determine the prevalence of consumption of IFA supplements and to identify factors associated with the consumption of IFA supplements among pregnant women in Eswatini.

**Methods:**

A cross-sectional questionnaire survey was conducted among 330 pregnant women aged ≥ 18 years in their third trimester in Eswatini. Participants were recruited from eight purposively selected healthcare facilities from July 2019 to October 2019. Good consumption was defined as consuming all or almost all IFA supplements throughout pregnancy.

**Results:**

During the first trimester, 10.3 % of the participants consumed all or almost all IFA supplements. In the second and third trimesters, those who consumed all or almost all supplements were 37 and 39.7 %, respectively, for iron and 37.6 and 40.9 %, respectively, for folic acid. Barriers, including side effects, forgetfulness, safe previous pregnancies without IFA, others’ advice against consumption, IFA stock-outs, inability to meet transport costs, and inadequate supply of IFA tablets, contribute to low consumption of IFA. Multivariate logistic regression models showed that the barriers were inversely associated with good consumption of IFA supplements. Better knowledge and attitude toward IFA and older maternal age were positively associated with good consumption of IFA supplements.

**Conclusions:**

Low consumption of IFA supplements in overall pregnancy is mainly owing to the late antenatal care attendance. Strategies such as establishing a preconception care unit and school-based provision of IFA may be helpful. It is evident that most women still lack knowledge, and some have negative attitudes about IFA supplements. Health education to raise awareness and emphasize the importance of starting antenatal care early as well as consuming supplements on time should be revisited and intensified. Multiple strategies such as including community health care workers for distributing IFA supplements, discussing with clients about the measures to reduce forgetfulness, advising ways to prevent and manage the side effects, providing subsidies to cover transport costs, and ensuring adequate supply of IFA supplements in facilities may need to be employed to reduce the identified barriers.

## Background

Nutritional requirements increase during pregnancy for supporting fetal growth and development as well as for maternal metabolism and tissue development specific to reproduction [1]. The recommended dietary allowance for iron and folic acid (IFA) for non-pregnant women is 18 mg and 400 µg [2] when compared with 27 mg and 600 µg per day for pregnant women [3], respectively. If the required increase in dietary allowance is not met, numerous undesirable conditions may manifest. Folate deficiencies have been linked to the development of neural defects in infants [4], and iron deficiencies have been linked to anemia and other conditions.

The average prevalence of anemia among pregnant women in developing countries is 56 %, which is significantly higher than that of 18 % in developed countries. In Africa, the prevalence of anemia in pregnant women is 48.2 % [5]. In Eswatini (a country in Southern Africa), 31 % of pregnant women fall under the classification of maternal anemia [6], although the possible reasons for the lower prevalence in Eswatini when compared with other developing countries have not been studied. Anemia is one of the major causes of pregnancy complications, leading to adverse maternal and perinatal outcomes, including maternal and perinatal mortalities [7]. Anemia in pregnancy is highly associated with the risk of miscarriages, stillbirths, prematurity, and low birth weight [8]. It is estimated that 40 % of women in Eswatini are likely to be anemic, which may be owing to insufficient iron [9]. It is also estimated that 300,000 babies are born each year with neural tube defects globally, and 15–20 % of all births are low birth weight. The 2014 Swaziland Multiple Indicator Cluster Survey indicated that 8 % of infants had low birth weight [10]. To prevent and cure these outcomes, the World Health Organization (WHO) strongly recommends daily oral supplementation of IFA as part of antenatal care (ANC). Studies conducted in different countries have shown that the use of IFA early and during one’s pregnancy can reduce undesirable pregnancy outcomes [11–13]. In Eswatini, the ANC package, which includes the provision of IFA, has been made accessible and affordable to every pregnant woman.

Despite the WHO recommendation, the use of IFA is still low in some developing countries. Adherence to iron supplementation across 22 Sub-Saharan African countries was 28.7 %, with some countries having low adherence, such as 1.4 % in Burundi [14]. Another study in Northern Tanzania revealed that the prenatal intake of folic acid and iron supplements by pregnant women was 17.2 and 22.3 %, respectively [15]. Nonetheless, the consumption of IFA was reported to be 93 % in South Africa [16]. To the best of our knowledge, there is no prior study on the consumption of IFA supplements among pregnant women in Eswatini.

The factors associated with poor consumption in countries such as Ethiopia and Egypt include lack of IFA supplements owing to not getting enough supplements to last until the next visit, forgetfulness, side effects of oral medications, little knowledge about the importance of supplements, and poor knowledge of anemia in pregnancy [8, 17]. In Eswatini, the problem of medication stock-outs is also common. At some point, the country’s local daily newspaper published that the country had run out of essential drugs for pregnant women [18].

According to several studies, the following factors are associated with good IFA consumption: more ANC visits, early ANC booking, counseling and education on IFA supplements, higher levels of education, diagnosis of anemia in a previous ANC visit, exposure to mass media, and presence of husbands during ANC visit [19–22]. Poor consumption arises not only because of the client’s behaviors but also because of factors outside the client’s control [23]. Such factors may include inadequate support from significant others and/or families and insufficient service delivery. Other client factors may include frustrations about the number and frequency of the pills, fear of macrosomia, etc. [24]. Limited information has been gathered regarding the level of consumption of IFA and related factors in Eswatini. Thus, this makes such a study a necessity. This study aimed to determine the consumption of IFA supplements, to explore IFA-related barriers, knowledge, and attitude, and to determine the factors associated with the consumption of IFA supplements among pregnant women in Eswatini.

## Methods

### Setting

The study was conducted in eight health care facilities that were selected to represent the four regions of Eswatini; each region was represented by two facilities. The facilities were Mbabane Government Hospital and Lobamba Clinic in the Hhohho region, Raleigh Fitkin Memorial Hospital and Family Life Association of Swaziland in the Manzini region, Sithobela Health Centre (Maternity unit) and Sithobela Public Health Unit in the Lubombo region, and Hlathikhulu Public Health Unit and Hlathikhulu Hospital (maternity unit) in the Shiselweni region. The eight health facilities were selected purposively, as these institutions provide ANC services and have high volumes of clients in the regions.

### Study design and participants

This study used a cross-sectional questionnaire survey design. The inclusion criteria were pregnant women aged ≥ 18 years, residing in Eswatini, in their third trimester, and visiting for a subsequent ANC at the eight selected health facilities. Participants < 18 years were not included because they needed parental consent to participate. We selected third-trimester pregnant women because we were interested in comparing IFA consumption across different trimesters. It seems to be easier for third-trimester pregnant women to recall their IFA consumption than postpartum women. Besides, a high use rate of any antenatal care (97.3 %) was reported in Eswatini [25]. Therefore, we expected a high coverage rate of antenatal care among third-trimester pregnant women. During the period from July 2019 to October 2019, 330 women were invited to participate in the study; they all agreed to participate.

### Ethics approval and consent to participate

 This study was approved by the National Health Research Review Board, Ministry of Health, in Eswatini (SRH139/2019). All procedures performed in studies involving human participants were in accordance with the 1964 Helsinki declaration and its later amendments or comparable ethical standards. Verbal and written informed consents were obtained from all participants.

### Sample size determination and sampling procedure

The sample size was determined using Cochran’s Sample Size Formula following these assumptions: 95 % significance level, 5 % margin of error, consumption of supplements from a previous survey being 70 % according to the last Demographic and Health Survey conducted in the country [9]. The calculated sample size provided a total of 323 participants. Overall, 330 participants were recruited over a period of 12 weeks, and each regional sample size was proportionate to the estimated number of ANC service users in the regions.

### Data collection tool

A researcher-developed structured questionnaire was used as the study tool. The tool was developed in English, later translated to Swazi, and back to English to ensure consistency. After developing the questionnaire based on the literature review, two experts in the field of maternal and child health from Eswatini, who were well informed about the local context, reviewed the questionnaire and gave feedback. The tool was pre-tested at Nhlambeni Clinic in the Manzini region to ensure that pregnant women could understand the questions without difficulty.

#### Variables measurement

The dependent variable, which was the consumption of IFA, was categorized into two categories: good and poor. Consumption of IFA was self-reported. Participants were asked if they took the supplements received in each of the three trimesters, and their responses were as follows: took all/almost all, took more than half, took half, took less than half, took a few, did not take, and not applicable (for participants who did not receive the supplements). Consuming all or almost all was categorized as good, and all the other response options were categorized as poor in each trimester. For overall consumption, participants were categorized as a good consumer if they had good consumption in all three trimesters and as a poor consumer if they had poor consumption in at least one trimester. Independent variables included socio-demographic characteristics (see Table 1 for the list of variables), knowledge, attitude, partner’s support, and barriers to IFA consumption. Partner’s support was measured using the question, “Do you receive support other than money from your spouse/partner?” with a five-point Likert scale (never, almost never, sometimes, almost always, every time).

**Table 1:** Participants’ characteristics based on overall consumption of iron and folic acid during pregnancy (*N* = 330)

**Characteristics**		Total			Iron			Folic acid		
		**n (%)**			**Good** **n (%)**	**Poor ** **n (%)**		**Good** **n (%)**		**Poor** **n (%)**
**Age, years**						*				
18–24		139	(42.1)		3 (13.6)	136 (44.2)		6 (23.1)		133 (43.8)
25–30		101	(30.6)		10 (45.5)	91 (29.5)		10 (38.5)		91 (29.9)
≥ 31		90	(27.3)		9 (40.9)	81 (26.3)		10 (38.5)		80 (26.3)
**Marital status**										
Single		167	(50.6)		8 (36.4)	159 (51.6)		10 (38.5)		157 (51.6)
Married but not living together		31	(9.4)		4 (18.2)	27 (8.8)		5 (19.2)		26 (8.6)
Married and living together		132	(40.0)		10 (45.5)	122 (39.6)		11 (42.3)		121 (39.8)
**Education**						**				**
Primary and below		69	(20.9)		3 (13.6)	66 (21.4)		3 (11.5)		66 (21.7)
Secondary/high school		214	(64.8)		9 (40.9)	205 (66.6)		14 (53.8)		200 (65.8)
Tertiary		47	(14.2)		10 (45.5)	37 (12.0)		9 (34.6)		38 (12.5)
**Religion**										
Christian		288	(87.3)		17 (77.3)	271 (88.0)		20 (76.9)		268 (88.2)
Other/non-religious		42	(12.7)		5 (22.7)	37 (12.0)		6 (23.1)		36 (11.8)
**Income**										
Sufficient/very sufficient		130	(39.4)		13 (59.1)	117 (38.0)		12 (46.2)		118 (38.8)
Barely sufficient		71	(21.5)		4 (18.2)	67 (21.8)		6 (23.1)		65 (21.4)
Insufficient/very insufficient		129	(39.1)		5 (22.7)	124 (40.3)		8 (30.8)		121 (39.8)
**Employment status**						*				
Unemployed		234	(70.9)		9 (40.9)	225 (73.1)		14 (53.8)		220 (72.4)
Self employed		28	(8.5)		3 (13.6)	25 (8.1)		3 (11.5)		25 (8.2)
Employed		68	(20.6)		10 (45.5)	58 (18.8)		9 (34.6)		59 (19.4)
**Place of residence**										
Urban		111	(33.6)		11 (50.0)	100 (32.5)		13 (50.0)		98 (32.2)
Rural		219	(66.4)		11 (50.0)	208 (67.5)		13 (50.0)		206 (67.8)
**Gravidity**										
Primigravidity		114	(34.5)		9 (40.9)	105 (34.1)		11 (42.3)		103 (33.9)
Multi/Grand-multi gravidity		216	(65.5)		13 (59.1)	203 (65.9)		15 (57.7)		201 (66.1)
**Gestational age at first ANC**						**				*
0–12 weeks		75	(22.7)		22 (100)	53 (17.2)		26 (100.0)		49 (16.1)
13–20 weeks		148	(44.8)		0 (0.0)	148 (48.1)		0 (0.0)		148 (48.7)
> 20 weeks		107	(32.4)		0 (0.0)	107 (34.7)		0 (0.0)		107 (35.2)
**Having a supportive partner**						*				*
Never/rarely		38	(11.5)		0 (0.0)	38 (12.3)		0 (0.0)		38 (12.5)
Sometimes		44	(13.3)		0 (0.0)	44 (14.3)		1 (3.8)		43 (14.1)
Always/almost always		248	(75.2)		22 (100.0)	226 (73.4)		25 (96.2)		223 (73.4)
					**Mean (SD)**	**Mean (SD)**		**Mean (SD)**		**Mean (SD)**
**Attitudes**					22.45(1.77)	19.07(3.11)		21.88(1.97)		19.07 (3.14)
**Barriers**					0.86 (1.05)	2.02 (1.08)		0.65 (0.69)		1.56 (1.22)
**Knowledge**					8.23 (3.27)	5.46 (2.81)		7.92 (3.12)		5.45 (2.81)

*indicates *p* values < 0.05 and ** indicates *p* values < 0.001 from chi-squared tests. *ANC* antenatal care; *SD* standard deviation

#### Knowledge

Participants were asked to list three sources of iron and three sources of folate, to state the correct timing of starting IFA supplementation, and to list three benefits of taking IFA supplements and three consequences of not taking IFA supplements during pregnancy. An individual’s responses to all the questions were added to form a sum of knowledge scores. The score ranged from 0 to 13, with a higher score indicating better knowledge. Internal consistency assessed using Cronbach’s alpha was 0.82.

#### Attitudes

The attitudes were assessed using five items with a Likert scale ranging from 1 to 5. Positive statements were coded as 1 for strongly disagree and 5 for strongly agree, and negative statements were reversely coded. Items were then added to compute a sum of scores, with a score ranging from 5 to 25, with a higher score indicating more positive attitudes toward IFA supplementation. Internal consistency assessed using Cronbach’s alpha for the attitude scale was 0.63.

#### Barriers

Participants were asked about their reasons for not having a 100 % IFA consumption in their pregnancy. Different reasons (10 reasons) for not consuming IFA were listed, and these reasons were summed up to compute a barrier score. The sum of scores ranged from 0 to 10, with a higher score indicating more barriers for that participant.

### Data analyses

Data analyses were performed using SPSS version 24 (IBM Corp, Armonk, NY). Descriptive analysis was performed to determine the prevalence of consumption of IFA supplements. The distribution of variables was evaluated using means and their standard deviations for continuous variables and proportions for categorical variables. Multivariate logistic regression was used for determining factors associated with the consumption of IFA supplements, with the odds ratio (OR) as the measure of association. A two-sided p-value of < 0.05 was considered statistically significant. For the selection of the model, the Akaike information criterion (AIC) was used, and the smallest AIC value represented a better fitting model. We used a stepwise method and started model building by entering all variables in the model, then dropping one variable with the greatest p-value at each step until all remaining variables were significant in the model. Goodness of fit of the final model was examined by the Hosmer-Lemeshow test (*p* > 0.05).

## Results

### Participants’ characteristics

Of the 330 participants, a majority (42.1 %) aged 18–24 years, 50.6 % of the participants reported being single on their marital status, 70.9 % were unemployed, and more than half (66.4 %) resided in rural areas. Less than half (34.5 %) of them were primigravidas. Only 22.7 % of the participants started their ANC visits within their first trimester, and 11.5 % of the participants reported that they rarely or never received support from their partners (Table 1).

### Consumption of IFA supplements

The consumption of IFA supplements by pregnant women is presented in Table 2. Good consumption was when the respondent consumed all or almost all supplements. During the first trimester, a few respondents (10 %) consumed all or almost all IFA supplements. In the second and third trimesters, those who consumed all or almost all supplements were 37 and 39.7 %, respectively, for iron and 37.6 and 40.9 %, respectively, for folic acid, which shows an increase in the consumption of both supplements from the first trimester. Women who did not receive IFA supplements owing to a lack of access were classified as not applicable (N/A). N/A in the first trimester was mainly because participants did not attend ANC in the first trimester; N/A in the second and third trimesters was owing to a lack of supply, as some facilities could not supply, and other participants were also failing to attend ANC on the dates they were booked for. When observing the overall consumption, those respondents who consumed supplements as required (i.e., all or almost all) in all three trimesters, the consumption was found to be good at 6.7 and 7.9 % for iron and folic acid supplements, respectively.

**Table 2:** Consumption of iron and folic acid supplements over the three trimesters of pregnancy (*N* = 330)

	Consumption of iron			Consumption of folic acid		
	**1st trimester**	**2nd trimester**	**3rd trimester**	**1st trimester**	**2nd trimester**	**3rd trimester**
All/almost all	34 (10.3)	122 (37.0)	131 (39.7)	34 (10.3)	124 (37.6)	135 (40.9)
More than half	15 (4.5)	67 (20.3)	67 (20.3)	14 (4.2)	57 (17.3)	69 (20.9)
Half	7 (2.1)	38 (11.5)	38 (11.5)	4 (1.2)	39 (11.8)	33 (10.0)
Less than half	7 (2.1)	32 (9.7)	36 (10.9)	10 (3.0)	32 (9.7)	33 (10.0)
Did not consume or consumed a few	6 (1.8)	23 (7.0)	40 (12.1)	9 (2.7)	31 (9.4)	43 (13.0)
N/A	261 (79.1)	48 (14.5)	18 (5.5)	259 (78.5)	47 (14.2)	17 (5.2)

N/A is not applicable, meaning women who cannot access iron and folic acid supplements. N/A refers to participants who did not attend antenatal care or did not receive the supply in facilities owing to limited stock

### Factors associated with the consumption of IFA

#### Barriers

There was a wide range of reasons for not consuming IFA (Table 3). The most frequently cited reasons were side effects (27.5 %) and forgetfulness (25.2 %). The mean number of barriers reported was 1.49 (standard deviation (SD), 1.21). Women who reported good consumption of IFA reported fewer barriers than women who reported poor consumption (mean (SD): 0.86 (1.05) vs. 2.02 (1.08) for iron; 0.65 (0.69) vs. 1.56 (1.22) for folate; both *p* < 0.001).

**Table 3:** Participants’ reasons for not consuming iron and folic acid supplements (N = 330)

Reasons	n	%
Long walking distance to health facility	27	8.2
Transport costs	40	12.1
Safe previous pregnancies without iron and folic acid supplements	56	17.0
Advised by friends/relatives not to consume	54	16.4
Use of traditional medicine	34	10.3
Forgetfulness	83	25.2
Side effects	89	27.0
Inadequate supply^*a*^	34	10.3
Tablets were out of stock in facility	47	14.2
Dislike medication	27	8.2
Number of barriers; Mean (SD)	1.49 (1.21)	

Number of barriers score range: 0–10. A high total score indicates more barriers. ^a^Inadequate supply: cases where participants were supplied with pills not covering the period up to next visit. *SD* standard deviation

#### Knowledge

Participants had limited knowledge regarding IFA (Table 4). When asked to name three food sources of iron and folic acid, 61.5 % of respondents knew at least two food sources of iron, while only 23.9 % of respondents knew at least two food sources of folate. When asked about the correct time to start taking IFA supplements, only 35.8 % responded correctly. When asked about the three benefits of taking the supplements, approximately 38.2 % of the respondents gave at least two correct benefits. For the consequences of not taking IFA supplements during pregnancy, only 21.6 % could give at least two correct consequences. The mean knowledge score was 5.65 (2.91). Women who reported good consumption of IFA reported a higher knowledge than those who reported poor consumption (mean (SD): 8.23 (3.27) vs. 5.46 (2.81) for iron; 7.92 (3.12) vs. 5.45 (2.81) for folate; both *p* < 0.001).

**Table 4:** Distribution of participants’ responses about knowledge (*N* = 330)

		Total		Iron consumption		Folate consumption	
**Variables**		**n**	**(%)**	**Good (*n* = 22)**	**Poor (*n* = 308)**	**Good (*n*** **= 26)**	**Poor (*n* = 304)**
A list of three food items that are a good source of iron.							
All three responses correct		91	(27.6)	10 (45.5)	81 (26.3)	11 (42.3)	80 (26.3)
Only two correct responses		112	(33.9)	9 (40.9)	103 (33.4)	10 (38.5)	102 (33.6)
Only one correct response		91	(27.6)	2 (9.1)	89 (28.9)	4 (15.4)	87 (28.6)
No correct response		36	(10.9)	1 (4.5)	35 (11.4)	1 (3.8)	35 (11.5)
A list of three food items that are a good source of folate.							
All three responses correct		36	(10.9)	4 (18.2)	32 (10.4)	5 (19.2)	31 (10.2)
Only two correct responses		43	(13.0)	3 (13.6)	40 (13.0)	4 (15.4)	39 (12.8)
Only one correct response		186	(56.4)	11 (50.0)	175 (56.8)	13 (50.0)	173 (56.9)
No correct response		65	(19.7)	4 (18.2)	61 (19.8)	4 (15.4)	61 (20.1)
When should a pregnant woman start consuming IFA supplements?							
Correct response		118	(35.8)	16 (72.7)	102 (33.1)	18 (69.2)	100 (67.1)
Incorrect response		212	(64.2)	6 (27.3)	206 (66.9)	8 (30.8)	204 (32.9)
A list of three benefits of consuming IFA supplements during pregnancy.							
All three responses correct		34	(10.3)	9 (40.9)	25 (8.1)	8 (30.8)	26 (8.6)
Only two correct responses		92	(27.9)	5 (22.7)	87 (28.2)	9 (34.6)	83 (27.3)
Only one correct response		146	(44.2)	6 (27.3)	140 (45.5)	7 (26.9)	139 (45.7)
No correct response		58	(17.6)	2 (9.1)	56 (18.2)	2 (7.7)	56 (18.4)
A list of three consequences of not consuming IFA supplements during pregnancy							
All three responses correct		19	(5.8)	8 (36.4)	11 (3.6)	7 (26.9)	12 (3.9)
Only two responses correct		52	(15.8)	6 (27.3)	46 (14.9)	7 (26.9)	45 (14.8)
Only one correct response		185	(56.1)	7 (31.8)	178 (57.8)	11 (42.3)	174 (57.2)
No correct response		74	(22.4)	1 (4.5)	73 (23.7)	1 (3.8)	73 (24.0)
Knowledge scores; Mean (SD)		5.65 (2.91)					

Score range: 1–13. A higher score indicates better knowledge. *IFA* iron and folic acid supplements; *SD* standard deviation

#### Attitude

Participants’ attitudes are presented in Table 5. The mean attitude score was 19.29 (3.16). Over 40 % (43.1 %) of women strongly agreed or agreed that “consuming IFA could result in macrosomia”, and about 10 % (9.1 %) of women strongly agreed or agreed that IFA should be consumed only when there is a problem. Those women who reported good consumption of IFA reported better attitude than those who reported poor consumption (mean (SD): 22.45 (1.77) vs. 19.07 (3.11) for iron; 21.88 (1.97) vs. 19.07 (3.14) for folate; both *p* < 0.001).

**Table 5:** Distribution of participants’ responses about attitude (*N* = 330)

Attitudes	Strongly agree	Agree	Not sure	Disagree	Strongly disagree
	**n (%)**	**n (%)**	**n (%)**	**n (%)**	**n (%)**
IFA is good for fetus	134 (40.6)	179 (54.2)	17 (5.2)	0 (0.0)	0 (0.0)
Pregnant women should adhere to IFA	122 (37.0)	112 (33.9)	90 (27.3)	6 (1.8)	0 (0.0)
Consuming IFA could result in fetal macrosomia	85 (25.8)	57 (17.3)	94 (28.5)	28 (8.5)	66 (20.0)
IFA could result in unsuccessful pregnancies	4 (1.2)	4 (1.2)	73 (22.1)	74 (22.4)	175 (53.0)
IFA should be taken only when there is a problem	6 (1.8)	24 (7.3)	92 (27.9)	105 (31.8)	103 (31.2)
Attitudes’ sum of score; Mean (SD)	19.29 (3.16)				

Score range: 0–25. A higher total score indicates more positive attitude towards IFA supplementation. *IFA* iron and folic acid; *SD* standard deviation

### Bivariate analysis

The bivariate analysis of different variables with the overall consumption status is presented in Table 1. The status of iron consumption in all trimesters significantly differed by age, education, and employment status. However, the status of folic acid consumption significantly differed by education and gestational age at the first ANC visit. Participants with good consumption of both iron and folic acid started attending ANC within the first 12 weeks of their pregnancy. Out of the participants who were classified as poor consumers of iron, only 17.2 % started attending ANC within the first 12 weeks of their pregnancy. From the poor consumers of folic acid, only 16.1 % started attending ANC within the first 12 weeks of their pregnancy. Participants with good consumption of IFA were significantly more likely to report that their partner was always or almost always supportive than participants with poor consumption (100 % vs. 73.4 % for iron; 96.2 % vs. 73.4 % for folic acid).

### Multivariate analysis

The parsimonious logistic regression model is presented in Table 6. The likelihood of consuming iron over the three trimesters increased with age. Using the age of 18–24 years as a comparison group, the odds of iron consumption for those aged 25–30 years were 2.92 (95 % confidence interval (CI): 0.71–12.06), and the odds for the group aged above 30 years were 4.44 (95 % CI: 1.09–18.10). An increase in the score of IFA knowledge was associated with good iron consumption (adjusted OR (AOR): 1.23, 95 % CI: 1.05–1.44), and similarly, an increase in the score of attitudes was associated with good iron consumption (AOR: 1.41, 95 % CI: 1.13–1.75). Having barriers was a significant factor, and as the score of barriers increased, the likelihood of consuming iron decreased (AOR: 0.53, 95 % CI: 0.29–0.97).

**Table 6:** Logistic regression models of factors associated with good IFA consumption during pregnancy (*N* = 330)

Good iron consumption				Good folic acid consumption		
**Explanatory variables**	**AOR**	**95 % CI**	***p*-value**	**AOR**	**95 % CI**	***p*-value**
**Age**				NS		
18–24	1.00					
25–30	2.92	(0.71–12.06)	0.14			
31 and above	4.44	(1.09–18.10)	0.04^*^			
**Knowledge**	1.23	(1.05–1.44)	0.001^*^	1.24	(1.07–1.43)	0.004^*^
**Attitude**	1.41	(1.13–1.75)	< 0.002^*^	NS		
**Barriers**	0.53	(0.29–0.97)	0.04^*^	0.51	(0.31–0.89)	0.02^*^

*NS* not statistically significant and was not included in the model; *IFA* iron and folic acid; *AOR* adjusted odds ratio; *CI* confidence interval. *P < 0.05

Table 6 illustrates that when a similar analysis was performed for folic acid consumption in the three trimesters, having a unit increase in the total score of knowledge about IFA was significantly associated with good consumption of folic acid supplements in the three trimesters (AOR: 1.24, 95 % CI 1.07–1.43). Table 6 also demonstrates that having barriers was a significant factor, and as the score of barriers increased, the likelihood of consuming folic acid decreased (AOR: 0.51, 95 % CI: 0.31–0.89). Attitude lost its statistical significance when knowledge and barriers were in the model for folic acid.

## Discussion

Our results showed that the overall good consumption of IFA supplements throughout pregnancy in Eswatini was approximately 7 and 8 % for iron and folic acid supplements, respectively. The overall adherence rate to iron supplementation across 22 Sub-Saharan African (SSA) countries (excluding Eswatini) was 28.7 % and ranged from 1.4 % in Burundi to 73.0 % in Senegal [14]. This consumption pattern is still relatively low than that in other countries, such as South Africa, where compliance with the consumption of IFA supplements was reported to be 93 % among pregnant women [26]. Another African country with a higher consumption rate is Kenya, with compliance to IFA at approximately 80 % [23]. The adherence to IFA supplementation was 28.7 % in Ethiopia [24], which was similar to this study in which the consumption was < 50 %. It was noted that the numbers might not be directly comparable since the measurement of adherence or good consumption varied across studies. Nonetheless, IFA supplementation for pregnant women in Eswatini needs to be further advocated.

Despite the low rate of good consumption of IFA supplementation throughout all trimesters of pregnancy, we found that the rate of good consumption increased from 10 % in the first trimester to approximately 40 % in the second and third trimesters. The low rate of good consumption of IFA supplements throughout pregnancy was mainly owing to late ANC booking, and thus, late commencement of IFA consumption since only 23 % of the participants booked ANC within their first trimester. It was noted that approximately 14 and 5 % of participants did not adhere to the consumption of IFA supplements in the second and third trimester, respectively, because they did not attend ANC or did not receive the supply in facilities due to limited stock. IFA consumption increased to 46 % in our participants during the second and third trimesters after those who did not receive IFA were excluded (data not shown). Thus, ANC in the first trimester should be emphasized, and supplies of IFA supplements in facilities should be better managed.

Participants’ age was found to be associated with iron consumption in our study, which is consistent with other studies [14, 26]. Ba and colleagues suggested that the positive association between age and adherence to iron supplementation during pregnancy could be explained by older women’s understanding of the benefits of iron supplementation for preventing anemia or having previously experienced iron deficiency-related adverse outcomes [14].

A positive association was observed between positive attitudes and consumption of IFA in our study. Strong agreement that IFA supplementation is good for the fetus was observed in 40.6 % of the participants, whereas negative attitude, such as strong agreement that IFA supplementation results in fetal macrosomia, was observed in 25.8 % of the study participants. Similarly, the beliefs that the supplement tablets would harm the baby were found to be significantly associated with non-compliance to IFA consumption in the Mecha district, Ethiopia [27]. The major barrier, associated with the lower consumption of IFA supplements, was the side effects, which was also listed in other studies as a major obstacle for the consumption and compliance [28, 29]. Therefore, it is recommended that healthcare workers in the facilities correct misconceptions about IFA consumption and instruct women on how to manage the potential side effects of IFA supplementation.

In our study, knowledge of IFA supplements was significantly associated with the consumption of folic acid supplements in the second and third trimesters. A similar finding was observed in Kenya, where the authors concluded that knowledge of IFA among pregnant women was associated with improved compliance [30]. Knowledge was significantly associated with compliance to prenatal IFA supplementation among women in Mecha, Ethiopia [27]. In another study in Northwest Ethiopia, knowledge about the benefits of supplements was associated with IFA supplementation adherence among pregnant women [31]. Based on the previous literature and our findings, we assume that informed pregnant women are more likely to consume adequate supplementation because they have been empowered, and they understand the benefits that come with supplementation.

Our study results should be acknowledged within the following limitations. The selection bias was possible, since the study participants were pregnant women attending antenatal care at eight healthcare facilities, which is not a random sample of pregnant women and may not represent pregnant women in Eswatini. Those women who used antenatal care may be more likely to take IFA than those who did not. Owing to the cross-sectional nature of the study, the associated factors were correlational rather than causal. The exclusion of women aged < 18 years limited the generalizability of our findings to all pregnant women and prevented the identification of factors that may be unique to this age group. However, these factors may be crucial when developing strategies to improve the consumption of IFA by all pregnant women. There is a possibility of recall bias since participants were asked about their consumption patterns from the first trimester to the third trimester. There is also a possibility that some of the participants were reporting what they deemed socially acceptable and reporting about pills that they did not ingest.

## Conclusions

In summary, IFA supplement consumption among pregnant women is poor in Eswatini, despite IFA supplementation being included as part of the ANC package. It is evident that most women still lack knowledge, and some have negative attitudes regarding IFA supplements. The findings show that side effects, forgetfulness, safe previous pregnancies without IFA, others’ advice against consumption, IFA stock-outs, inability to meet transport costs, and inadequate supply of IFA tablets are barriers to adherence to IFA supplement consumption. To improve consumption, strategies such as establishing a preconception care unit and school-based provision of IFA supplements may be helpful. Multiple strategies need to be employed to overcome the identified barriers. These strategies may include community health care workers for distributing IFA supplements, discussing with clients about the measures to reduce forgetfulness, advising ways to prevent and manage the side effects, providing subsidies to cover transport costs, and ensuring adequate supply of IFA supplements in facilities. To address the negative attitudes and lack of knowledge, known accurate information should be revisited and reinforced in health education, myths should be dispelled, and the importance of IFA supplementation should be intensified.

## Data Availability

Data is available upon reasonable request from the corresponding author.

## Abbreviations

IFA: Iron and folic acid
ANC: Antenatal care
WHO: World Health Organization
SD: Standard deviation
AOR: Adjusted odds ratio
AIC: Akaike information criterion
CI: Confidence interval
